# *FCGR* Genetic Variation in Two Populations From Ecuador Highlands—Extensive Copy-Number Variation, Distinctive Distribution of Functional Polymorphisms, and a Novel, Locally Common, Chimeric *FCGR3B/A* (CD16B/A) Gene

**DOI:** 10.3389/fimmu.2021.615645

**Published:** 2021-05-24

**Authors:** Manuela Moraru, Adriana Perez-Portilla, Karima Al-Akioui Sanz, Alfonso Blazquez-Moreno, Antonio Arnaiz-Villena, Hugh T. Reyburn, Carlos Vilches

**Affiliations:** ^1^ Immunogenetics & Histocompatibility Lab, Instituto de Investigación Sanitaria Puerta de Hierro—Segovia de Arana, Majadahonda, Spain; ^2^ Department of Immunology and Oncology, National Centre for Biotechnology (CNB-CSIC), Madrid, Spain; ^3^ Department of Immunology, School of Medicine, University Complutense, Madrid, Spain

**Keywords:** Fc gamma receptors, *FCGR* locus, CD16A, copy-number variation, copy number region, polymorphism, genetic variation, Ecuador

## Abstract

Fcγ receptors (FcγR), cell-surface glycoproteins that bind antigen-IgG complexes, control both humoral and cellular immune responses. The *FCGR* locus on chromosome 1q23.3 comprises five homologous genes encoding low-affinity FcγRII and FcγRIII, and displays functionally relevant polymorphism that impacts on human health. Recurrent events of non-allelic homologous recombination across the *FCGR* locus result in copy-number variation of ~82.5 kbp-long fragments known as copy-number regions (CNR). Here, we characterize a recently described deletion that we name CNR5, which results in loss of *FCGR3A*, *FCGR3B*, and *FCGR2C*, and generation of a recombinant *FCGR3B/A* gene. We show that the CNR5 recombination spot lies at the beginning of the third *FCGR3* intron. Although the *FCGR3B/A-*encoded hybrid protein CD16B/A reaches the plasma membrane in transfected cells, its possible natural expression, predictably restricted to neutrophils, could not be demonstrated in resting or interferon γ-stimulated cells. As the CNR5-deletion was originally described in an Ecuadorian family from Llano Grande (an indigenous community in North-Eastern Quito), we characterized the *FCGR* genetic variation in two populations from the highlands of Ecuador. Our results reveal that CNR5-deletion is relatively frequent in Llano Grande (5 carriers out of 36 donors). Furthermore, we found a high frequency of two strong-phagocytosis variants: the *FCGR3B*-NA1 haplotype and the CNR1 duplication, which translates into an increased *FCGR3B* and *FCGR2C* copy-number. CNR1 duplication was particularly increased in Llano Grande, 77.8% of the studied sample carrying at least one such duplication. In contrast, an extended haplotype CD16A-176V – CD32C-ORF+2B.2 – CD32B-2B.4 including strong activating and inhibitory FcγR variants was absent in Llano Grande and found at a low frequency (8.6%) in Ecuador highlands. This particular distribution of *FCGR* polymorphism, possibly a result of selective pressures, further confirms the importance of a comprehensive, joint analysis of all genetic variations in the locus and warrants additional studies on their putative clinical impact. In conclusion, our study confirms important ethnic variation at the *FCGR* locus; it shows a distinctive *FCGR* polymorphism distribution in Ecuador highlands; provides a molecular characterization of a novel CNR5-deletion associated with CD16A and CD16B deficiency; and confirms its presence in that population.

## Introduction

Most leukocyte lineages express one or more FcγR, receptors that bind the crystallisable fragment of IgG. FcγR constitute a family of proteins that connect humoral and cellular immune responses. By recognizing antigen-IgG complexes, they confer specificity to innate cellular immunity, and trigger, enhance or regulate the function of different cells of the immune system. FcγR recognition of soluble immune complexes, opsonized particles or aggregated IgG triggers a wide array of effector and regulatory responses including phagocytosis, antibody-dependent cellular cytotoxicity (ADCC), cytokine production, B cell homeostasis, immune complex clearance and antigen presentation. The complex regulation of immune responses mediated by FcγRs is further increased by genetic polymorphism.

**Figure 1:**
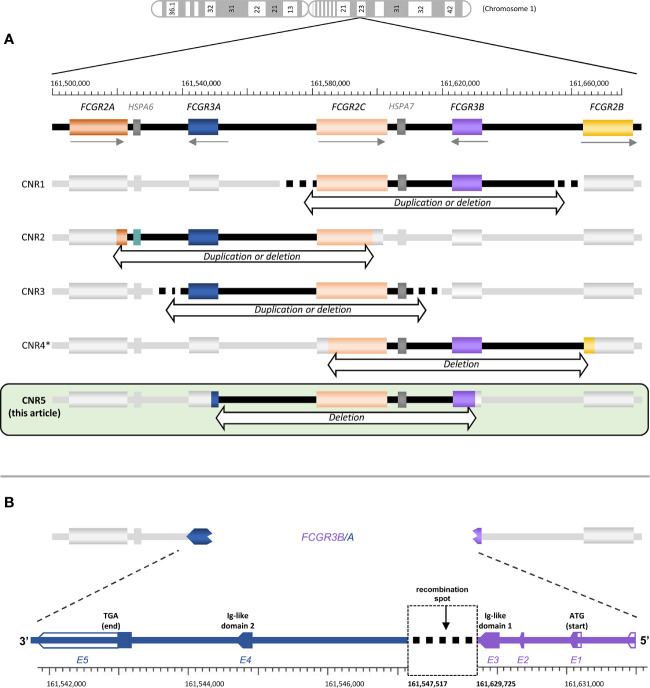
*FCGR* locus structure and variation. **(A)** The structure of the *FCGR* locus and the approximate localization of all known *FCGR* CNR are depicted, including the one described here. **(B)** Schematic representation of the *FCGR3B/A* recombination. Empty arrows correspond to untranslated regions. Gene locations and nucleotide numbering are derived from the human genome build GCRh38. *CNR4 represented here is the one described in Nagelkerke et al. (3). Lassauniere et al. (4) also called CNR4 a different region, involving exclusively *FCGR2C* (i.e., it does not cover a region of ~82.5 Kbp, compatible with NAHR event of the repeat units in the *FCGR* locus). Because its presence in the genome has not been confirmed by other studies, we have not included it here.

Human FcγR include both activating and inhibitory receptors of three major types that differ in structure, expression and affinity for IgG; FcγRI (or CD64), FcγRII (or CD32, including three structurally related receptors: CD32A, B and C) and FcγRIII (or CD16, comprising CD16A and B). FcγRII and FcγRIII bind immune complexes with lower affinity than FcγRI, and are encoded in the *FCGR* locus on chromosome 1q23.3 (whereas *FCGR1* is on 1q21.2). Because *FCGR* genes have evolved by duplication and unequal crossing-over, they show a high degree of structural homology and sequence identity (1). The complexity of the *FCGR* locus is further increased by recurrent events of non-allelic homologous recombination [NAH, (2)] that have given rise to copy-number variation (CNV) and, occasionally, chimeric genes (3). Four CNV regions of ~82.5 Kbp (designated CNRs), each of them subjected to deletion or duplication, have been defined (**Figure 1A**). CNR1 is most common and encompasses *FCGR2C* and *FCGR3B* (coding for CD32C and CD16B, respectively); CNR2 starts with exon 8 of *FCGR2A* (CD32A) and ends before *FCGR2C* (CD32C) exon 8, thus including the intervening *FCGR3A* gene (CD16A); CNR3 includes *FCGR3A* and *FCGR2C* and it is mainly found in Southeast Asian populations (5); and CNR4, possibly least common, spans from *FCGR2C* intron 3 to *FCGR2B* intron 3 (3). CNR4 duplication might actually be considered a variant of CNR1, from which it is indistinguishable with standard methods because of the high degree of homology between the two 82.5 kbp-long repeat units (3, 6, 7).

Even though CD32 and CD16 are classically designated as low-affinity receptors, avidity is quite variable – FcγR type and surface density, and IgG subclass and allotype, all modify the strength of receptor-immune complex interaction and its biological outcome. Moreover, FcγR sequence variation further modulates their function. In this regard, the functional consequences of genetic variations affecting the ligand-binding domains and transcriptional regulatory regions, and their relationship with disease, have all been extensively studied. The *FCGR3A*-p.Val176Phe replacement (rs396991, frequently referred as Val158Phe), which increases CD16A (FcγRIIIa) affinity for IgG (8), has been related to the clinical course of haematological, autoimmune and infectious diseases, and to monoclonal antibody therapy outcome (9–14). CD16A is expressed by most peripheral blood NK cells, as well as subsets of monocyte/macrophages, NKT and γδ T cells, and mediates cell activation, through the signalling adaptor molecules CD247 (CD3ζ) and FcϵR1γ. Immune complex engagement by CD16A triggers ADCC, a major effector mechanism of the immune response to infectious pathogens and malignant cells.

CD16B is expressed on polymorphonuclear cells, at particularly high levels on neutrophils. Since it is anchored to the plasma membrane by a glycosylphosphatidylinositol (GPI) moiety, CD16B is unlikely to signal, instead it is thought to function as a scavenger receptor, contributing to immune-complex clearance and modulating neutrophil-mediated ADCC potency (15, 16). Combinations of six SNPs determine three major *FCGR3B* haplotypes, NA1, NA2 and SH, that encode CD16B allotypes of the Human Neutrophil Antigen1 (HNA1). Of these, CD16B-HNA-1a, encoded by the *FCGR3B*-NA1 haplotype, has increased affinity for IgG3 and mediates stronger phagocytosis of IgG-opsonized particles compared to CD16B-HNA-1b (17, 18). Both *FCGR3B* CNV and its HNA-1a allotype have been related with the clinical course of autoimmune disease, particularly systemic lupus erythematosus (SLE) (14, 19).

CD32A (FcγRIIa), a single-chain transmembrane receptor carrying a non-classical immunoreceptor tyrosine-based activation motif in its cytoplasmic tail, is widely expressed on the myeloid lineage. Because it is the only human FcγR that binds IgG2, besides IgG1, 3 and 4, it is important for pathogen clearance (8). The *FCGR2A*-p.166His allotype (rs1801274, often referred to as *FCGR2A*-131His) significantly increases the CD32A-IgG2 interaction (8), being considered for many years as the sole FcγR capable of efficient interaction with IgG2 (20, 21). As expected, *FCGR2A*-p.166His has been associated with the clinical course of infectious and chronic inflammatory disease (15, 21–23).

The only inhibitory FcγR is CD32B, a single-chain receptor that modulates the activation threshold of a variety of leukocytes. The *FCGR2B*-p.Thr232Ile change (rs1050501) alters CD32B affinity for lipid rafts, thereby limiting its inhibitory function, and it seems to improve survival in malaria, while increasing susceptibility to SLE and childhood idiopathic thrombocytopenia (15, 24, 25). Two other SNPs of the *FCGR2B* (and *FCGR2C*) promoter define the *FCGR2B*-2B.4 haplotype [–386C, rs143796418, and –120A, rs780467580, in almost complete linkage disequilibrium (LD)], which associates with increased transcription and SLE (26–29).

*FCGR2C*, product of an ancestral unequal crossover between *FCGR2A* and *FCGR2B*, codes for CD32C, which is not expressed in most individuals due to common nonsense (i.e., *FCGR2C-*p.57Ter, rs759550223) and splice-site (e.g., rs76277413) mutations. Only about 20% of individuals of European origin carry a classic CD32C-ORF haplotype, defined by *FCGR2C-*p.57Gln and a correct splice site (c.798+1G), which allows for surface expression (30, 31). The *FCGR2C* and *FCGR2B* promoters are virtually identical, and they also share SNPs – whereas the 2B.4 haplotype (–386C, –120A) is restricted to *FCGR2B*, haplotype 2B.2 (–386C, –120T) is almost exclusively found in *FCGR2C* (29). The classic CD32C-ORF variant is in nearly complete LD with the 2B.2 promoter haplotype (31). Furthermore, close proximity within a ca. 200-Kbp locus favours LD between polymorphisms of different *FCGR* loci. For instance, variable strength of association has been reported between *FCGR2C*-ORF and the *FCGR2B*-2B.4 haplotype, *FCGR3A-*p.176Val, *FCGR2A*-p.166His and *FCGR2A*-p.67Trp, an SNP of unknown functional implication (4, 5, 31–33). However, LD across the *FCGR* locus could vary in different populations and deserves further attention.

We have recently reported on the first complete primary CD16A deficiency in several members of an Ecuadorian family, due to NAHR and CNV in the *FCGR* locus (34). Affected individuals carried a CNR2 deletion (affecting both *FCGR3A* and *FCGR2C*) on one chromosome; and a new deletion, which we name CNR5, on the other one. The CNR5 deletion affects parts of *FCGR3A* and *FCGR3B* and the intervening sequence, creating a *FCGR3B/A* fusion gene (**Figure 1A**). In the present study we provide a more in-depth characterization of this novel CNR. Furthermore, we assess the organization and genetic variation of the *FCGR* locus in two population samples from Ecuador highlands, one of them from Llano Grande, homeland of the family with CD16A deficiency.

## Materials and Methods

### Subjects and Samples

Thirty-six healthy individuals, original from the community of Llano Grande (Northeast of Quito in Ecuador highlands), were enrolled with kind help from *Asociación Llano Grande Deporte y Cultura* and *Asociación de mujeres de bordado*. They had all emigrated in recent years to Madrid, where they maintained a relatively closed community (35). All selected individuals were adults without first or second-degree family relationship between them or with previously reported CD16A-deficient individuals (34). After written informed consent, DNA and peripheral blood cells were extracted from venous blood using standard procedures. Flow cytometry studies were performed in eight additional individuals – the parents of the first cases of CD16A deficiency and six first-degree relatives of the 36 previously selected individuals. Genetic studies were also performed in an additional cohort of 70 healthy adults who had emigrated to Madrid from two different rural regions close to the cities of Quito and Cuenca, in Ecuador highlands (the *Sierra*), as previously described (36). The study was conducted according to the Declaration of Helsinki and was approved by the Ethics Committee of *Hospital Universitario Puerta de Hierro Majadahonda* (no. 13.18).

### Primary Cells and Cell Lines

Peripheral blood mononuclear cells (PBMC) and neutrophils were isolated from whole blood with a two-step density gradient as previously described (37). They were washed twice in HEPES-buffered saline (Ca^2+^ and Mg^2+^ free; Gibco Life Sciences) and resuspended at 10^6^ cells/mL in the same medium. For phosphoinositide phospholipase (PI-PLC) treatment, freshly isolated granulocytes were washed and resuspended in 25 mM Tris, pH 8, 150 mM NaCl before either mock digestion, or PI-PLC (Sigma Aldrich) treatment as described previously (38), for 1 h at 37°C. After washing, cells were stained to analyse CD16 and CD59 expression by flow cytometry.

The HEK-293T cell line was cultured in Dulbecco´s Modified Eagle medium (DMEM) supplemented with 10% foetal calf serum (FCS), 2 mM L-Glutamine, 0.1 mM sodium pyruvate, 100 U/mL penicillin, 100 U/mL streptomycin, 50 μM β-mercaptoethanol, maintained at 37°C and 5% CO_2_ in a humidified incubator and split as necessary. The HEK-293T cells were transfected, using the jetPEI transfection reagent (PolyPlus Transfection) with plasmids bearing the different CD16 constructs, with or without plasmids encoding the signalling adaptor molecules FcϵR1γ or CD247 (39). Twenty-four hours after transfection, cells were recovered for flow cytometry analysis or western blot assays.

### Genotyping

*FCGR*-cluster polymorphism was analysed using a multiplex ligation-dependent probe amplification (MLPA) method (MRC-Holland, Amsterdam, The Netherlands) that includes gene- and allele-specific probes allowing determination of the CNV and SNPs in all genes in this locus (40).

To identify the *FCGR3B/A* recombination spot, a genomic DNA fragment spanning the *FCGR3* third intron and fragments of the adjacent exons was first amplified by PCR in samples from CD16A-negative individuals using a forward oligonucleotide primer, 5’-ccctgaggacaattccacac-3’, specific for both *FCGR3A* and *FCGR3B*, and a reverse, *FCGR3A*-specific primer, 5’-ctttgagtgtggcttttggaatgta-3’. Verification sequences of a similar region were subsequently obtained from additional *FCGR3B/A* carriers (who bore canonical *FCGR3B* and *FCGR3A* genes on the other chromosome) using forward oligonucleotide 5’-cacacagtggtttcacaatgagaa-3’, specific for *FCGR3B/A* and *FCGR3B*-NA1, and the same reverse primer. Additional *FCGR3B*-NA1 sequences of the same region were obtained with forward 5’- cacacagtggtttcacaatgagaa -3’ and reverse 5’- cacattccaaaagccacactc -3’, in samples from *FCGR3B*-NA1/NA1 individuals. All PCRs were carried out with Advantage-2 polymerase (BD-Clontech, Palo Alto, CA, USA), 100 ng of genomic DNA and 1 μM of each oligonucleotide. PCR conditions were: initial denaturation for 2 min at 95°C; 10 cycles of 30 s at 95°C, 4 min at 72°C; 25 cycles of 30 s at 95°C, 30 s at 70°C and 4 min at 72°C; and 2 min at 72°C. These products were then sequenced using internal oligonucleotide primers and analysed in an ABI Prism 3100-Avant Genetic analyser (Applied Biosystems) in the central facilities of IDIPHISA. Nominal specificity of all oligonucleotides was verified a posteriori by ruling out presence of *FCGR3B* and *FCGR3A* sequences.

To screen for the CNR5 deletion, we used PCR with sequence-specific primers (SSP) 5’-cacacagtggtttcacaatgagaa-3’ (forward, specific for *FCGR3B*-NA1 and *FCGR3B/A*), and 5’-gaacagccgtggaagtagag-3’ (intronic, reverse, specific for *FCGR3A* and *FCGR3B/A*). Another primer pair recognizing non-polymorphic sequences of the *COCH* gene served as an internal positive control (IPC, 5’-gaaagaaacttgtgtgttgtctggt-3’ and 5’-attgggtaaagccacaggtgtttg-3’). PCR was performed using 100 ng genomic DNA, 1 U BioTaq polymerase (Bioline, London, UK), 0.67 μM specific primers and 1 μM IPC primers. PCR conditions were: 2 min at 95°C; 10 cycles of 10 s at 94°C, 30 s at 70°C and 90 s at 72°C; 20 cycles of 10 s at 94°C, 30 s at 67°C and 90 s at 72°C; and 2 min at 72°C.

### Nomenclature

In accordance with the guidelines of the Human Genome Variation Society, SNP nomenclature used along this manuscript refers to the amino acid positions in the full protein (41). **Table 1** describes the equivalence between different commonly used names for all studied SNPs along the *FCGR* locus. Nucleotide numbering is based on the human reference genome GRCh38 (hg38). *FCGR3* exon numbering refers to most abundant *FCGR3A* and *FCGR3B* transcripts (i.e., *FCGR3A1* and *FCGR3B1* – other common transcript variants include an additional exon in its 5’ end).

**Table 1:** Nomenclature of *FCGR* polymorphisms.

Receptor	Recommended name	Other common designations*	Nucleotide	Reference	Functional change
CD16A	*FCGR3A*-p.Val176Phe	*FCGR3A*-V158F	c.526T>G	rs396991	Affinity for IgG
CD16B	*FCGR3B*-NA1/NA2 haplotypes		c.108G>C, c.114C>T, c.194A>G, c.244G>A, c.316G>A	rs200688856, rs527909462, rs448740, rs147574249, rs2290834	Phagocytic activity
CD16B	*FCGR3B*-SH/NA2 haplotypes		c.233A>C	rs5030738	Protein tertiary structure
CD32A	*FCGR2A*-p.His166Arg	*FCGR2A*-H131R	c.497G>A	rs1801274	Affinity for IgG
CD32B	2B.4/2B.1 haplotypes		−386 C>G, −120 A>T	rs143796418, rs780467580	Protein expression
CD32B	*FCGR2B*-p.Thr232Ile		c.695T>C	rs1050501	Receptor inclusion in lipid rafts
CD32C	2B.2/2B.1 haplotypes		−386 C>G, −120 T	rs149754834, rs34701572	Unknown
CD32C	*FCGR2C*-p.Gln57Ter	*FCGR2C*-Q13X or *FCGR2C*-ORF/STOP	c.169T>C	rs759550223	Protein expression
CD32C	*FCGR2C* classic ORF haplotype		c.169C, c.798 +1G	rs759550223, rs76277413	Protein expression

*Numbering in the mature protein.

### Flow Cytometry

The following antibodies were used in flow cytometry experiments of whole blood: CD3-FITC (clone UCHT1), CD56-PE (clone HCD56), CD16-APC (clone 3G8), all from Biolegend, and CD59-APC (clone MEM-43) from ImmunoTools. Unconjugated CD16-specific monoclonal antibodies 3G8 and B73 (purified from hybridoma supernatants by affinity chromatography using Protein G-Sepharose (GE Healthcare), hybridomas were a gift of Prof JL Strominger), followed by goat anti-mouse secondary antibodies coupled to FITC (Dakocytomation) were used to stain the transfected HEK-293T cells (the two mAbs gave similar results). Unlabelled IgG1 and IgG2a isotype control mAbs were purchased from Sigma Aldrich, while fluorescently labelled isotype control antibodies were purchased from Biolegend.

Most experiments were carried out by staining 100 µl of whole blood with directly labelled mAb. Blood samples were washed in PBS buffer containing 0.5% (w/v) bovine serum albumin, 1% (v/v) foetal bovine serum, 0.1% sodium azide, and stained with labelled mAbs specific for the indicated receptors for 40 min at 4°C. Red blood cells were lysed with 1 ml VersalyseTM (Beckman Coulter) for 10 min before analysis in FACSCalibur (BD Biosciences) cytometer. Data were analysed with FlowJo and Kaluza Flow Cytometry Analysis programs.

### Western Blot

Cells were collected, washed once with ice-cold PBS and lysed in 50 mM Tris pH 7.4, 150 mM NaCl, 1% Triton X-100, 1% Sodium deoxycholate, 0.1% SDS, 1 μM Pepstatin A, 1 μM Leupeptin, and 0.5 mM Iodoacetamide for 30 min on ice. Lysates were centrifuged to pellet insoluble material for 15 min at 4°C. After quantitation, 25-30 μg protein was loaded onto 12% SDS polyacrylamide gels, electrophoresed under reducing conditions, and transferred to PVDF filters (Immobilon-P, Millipore). Membranes were blocked in 5% dried skimmed milk (1 h, 25°C), washed three times in 0.05% Tween/TBS and incubated (overnight, 4°C) in primary antibodies: mouse monoclonal anti-CD16 (clone DJ130c), mouse monoclonal anti-HLA B/C (clone HC10 (42), a gift of Prof. JA López de Castro), rabbit polyclonal anti CD247 (CD3ζ, a gift of Prof. B Alarcon) or rabbit polyclonal anti-FcϵR1γ (Merck Millipore) in 0.05% Tween/TBS. Membranes were then washed in 0.05% Tween/TBS (5 min, 2X) and bound antibodies were visualized using goat anti-mouse or goat anti-rabbit secondary reagents (DakoCytomation) and the ECL-Western Blot Detection Reagent (GE Healthcare). For N-glycanase treatment, 25 μg of sample lysate was denatured for 10 min at 100°C and treated with 0.25 units of PNGase-F (New England Biolabs) for 1 h at 37°C before analysis by SDS-PGE and western blot.

### Statistical Analysis

Gene frequencies were estimated by direct counting and they were compared using the Fisher exact test. Linkage disequilibrium between *FCGR* functional variants, and between CNRs and flanking SNPs, was assessed using standard procedures (43). An R script, kindly provided by Nathan E. Wineinger (44), was used to estimate allelic and haplotype frequencies of SNPs inside CNV regions (**Supplementary Table 3**). Linear regression test was performed to assess correlation between *FCGR3B* copy number and CD16B surface expression on granulocytes. A Mann-Whitney non-parametric test was used to compare CD16A MFI on NK cells between *FCGR3A* one- and two-copy carriers.

## Results

### Characterization of a Novel CNR in the *FCGR* Locus

Family studies of the first individuals with complete CD16A deficiency showed that one parental chromosome 1 carried a hybrid *FCGR3B/A* gene (34), whose 5’ exons appear to derive from a *FCGR3B* sequence carrying the NA1 haplotype. Here, we further dissect the break-point location, which, from cDNA sequencing and MLPA studies, should be located in the third, largest *FCGR3A/3B* intron, which separates the exons encoding the two CD16 Ig-like domains (**Figure 1B**). Because genomic *FCGR3B*-NA1 sequences were lacking, we first determined complete intron 3 sequences in three NA1-homozygous subjects (GeneBank accession nos. MW620867 and MW573976). Alignment of novel and previously available intron 3 sequences shows a high degree of identity between *FCGR3A* and *FCGR3B* variants. Moreover, less than ten SNPs distinguish *FCGR3A* and *FCGR3B* along the first 1000 bp, compared to more than one hundred differences in the rest of the intron. In fact, intron 3 of *FCGR3B*-NA1 is, in its first 904 bp, less similar to NA2 haplotype of the same gene than to its *FCGR3A* paralogue.

Sequence analysis of the same region in CNR5-deletion carriers (MW573977) showed that, starting from nucleotide 905, intron 3 of the hybrid *FCGR3B/A* matches exactly *FCGR3A* sequences deposited in EMBL/GenBank/DDBJ databases with accession nos. AL590385.12, NC0000011.11 and NG009066.1; whilst diverging from *FCGR3B* in, at least, 56 SNPs and 100 indels. The recombination spot thus lies between intron 3 nucleotides 53 and 904, but the high degree of identity between *FCGR3B*-NA1 and *FCGR3A* in that region precluded a more precise determination (**Figure 1B**).

### *FCGR3B/A* mRNA, but No Recombinant CD16B/A Protein, Is Detected in Granulocytes

**Figure 2:**
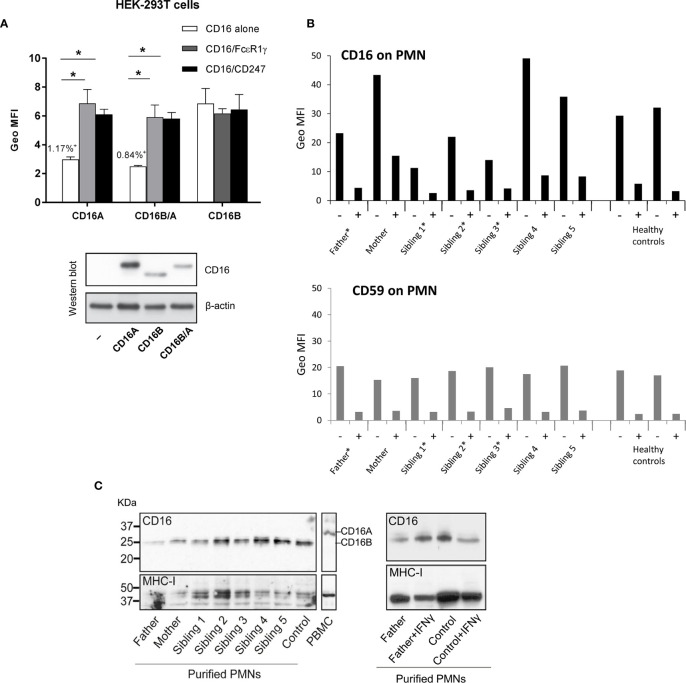
CD16B/A expression on polymorphonuclear and HEK-293T cells. **(A)** The transmembrane CD16BA receptor must associate with FcϵR1γ or CD247 for cell surface expression. HEK-293T cells were transfected with plasmids driving the expression of the indicated receptors and adaptor molecules and cell surface expression of CD16 was determined by flow cytometry and western blot. In all cases, more than 40% of the transfected cells expressed CD16 on the cell surface except for those cells transfected with CD16A or CD16B/A alone where CD16 staining was detected on only 1.17% and 0.84% of the cells, respectively. **(B)** No evidence for expression of a PI-PLC resistant form of CD16 on granulocytes of controls or family members. Freshly isolated granulocytes were mock digested, or PI-PLC treated, for 1hr at 37°C and then CD16 expression was analysed by flow cytometry. Asterisks mark *FCGR3B/A* carriers. **(C)** Western blot analysis of granulocyte lysates shows that the CD16 molecules present have a molecular weight typical of GPI-linked CD16B. Freshly isolated granulocytes were lysed and CD16 expression analysed, after treatment with PNGase, by western blot. On the right side, analyses of fresh and IFNγ-stimulated polymorphonuclear cells isolated from blood of a CNR5 carrier (father) and a normal control donor do not reveal induction of a *FCGR3B/A* product.

We previously determined by qRT-PCR that granulocytes express low levels of the novel recombinant *FCGR3B/A* mRNA, but no such transcripts were found in peripheral blood mononuclear cells of the same individual (34). This was consistent with our predicted expression pattern, as the recombinant gene carries the *FCGR3B* 5’ regulatory sequence that determines CD16B expression on polymorphonuclear leukocytes. We have now assessed whether a fusion CD16B/A protein is actually expressed on the cell surface. First, we found that, when overexpressed in HEK-293T cells, the recombinant *FCGR3B/A* gene isolated from the CD16A-deficient patient, like wild-type *FCGR3A*, is only expressed at the cell surface when co-transfected with the signalling molecules FcϵR1γ or CD247 (**Figure 2A**). This is in contrast to *FCGR3B*, which needs no adaptors for expression, because its product is anchored to the cell membrane by a GPI moiety. Next, to test for natural expression of the chimeric protein *ex vivo*, we exploited the fact that granulocyte CD16B can be cleaved by phosphoinositide phospholipase (PI-PLC) because it is GPI-anchored to the cell membrane (45). PI-PLC treatment should not affect surface expression of CD16B/A, as this receptor is predicted to have a transmembrane domain. We therefore incubated polymorphonuclear leukocytes of donors carrying or lacking *FCGR3B/A* with PI-PLC, which reduced CD16 expression in all donors tested. A similar reduction was also noted for a control GPI-anchored protein (CD59). In other words, after exposure to PI-PLC, no *FCGR3B/A* carrier retained significant CD16 surface expression on their granulocytes that could be attributed to a transmembrane form of this receptor (**Figure 2B**). Consistent with this data, western blot analysis of N-deglycosylated lysates of freshly-isolated and interferon γ-activated granulocytes failed to reveal a CD16B/A band, of (predicted) slower mobility than CD16B (**Figure 2C**). Taken together, our results suggest that CD16B/A could potentially reach the plasma membrane, but its expression in fresh or stimulated granulocytes is much lower than CD16B, not reaching levels detectable with the methods we have used.

### Unique Distribution of *FCGR* Genetic Variations in an Ecuadorian Population From Llano Grande

As the first complete CD16A deficiency was characterized in Ecuadorian individuals, and information on the organization and genetic variation of the *FCGR* locus in South American populations is yet insufficient, we sought to assess *FCGR* polymorphism in Ecuadorians. The patients with complete CD16A deficiency were native to Llano Grande, an indigenous community Northeast of Quito, Ecuador. We reasoned that, as their *FCGR3A* loss was the result of two different genetic events (CNR2 and CNR5 deletions), the frequency of these might be increased in this population. To test this hypothesis and characterize *FCGR* polymorphism, we used MLPA to study both CNV and SNPs in the *FCGR* locus of 36 healthy individuals from Llano Grande with no first- or second-degree family relationship between them or with the immunodeficient individuals.

**Figure 3:**
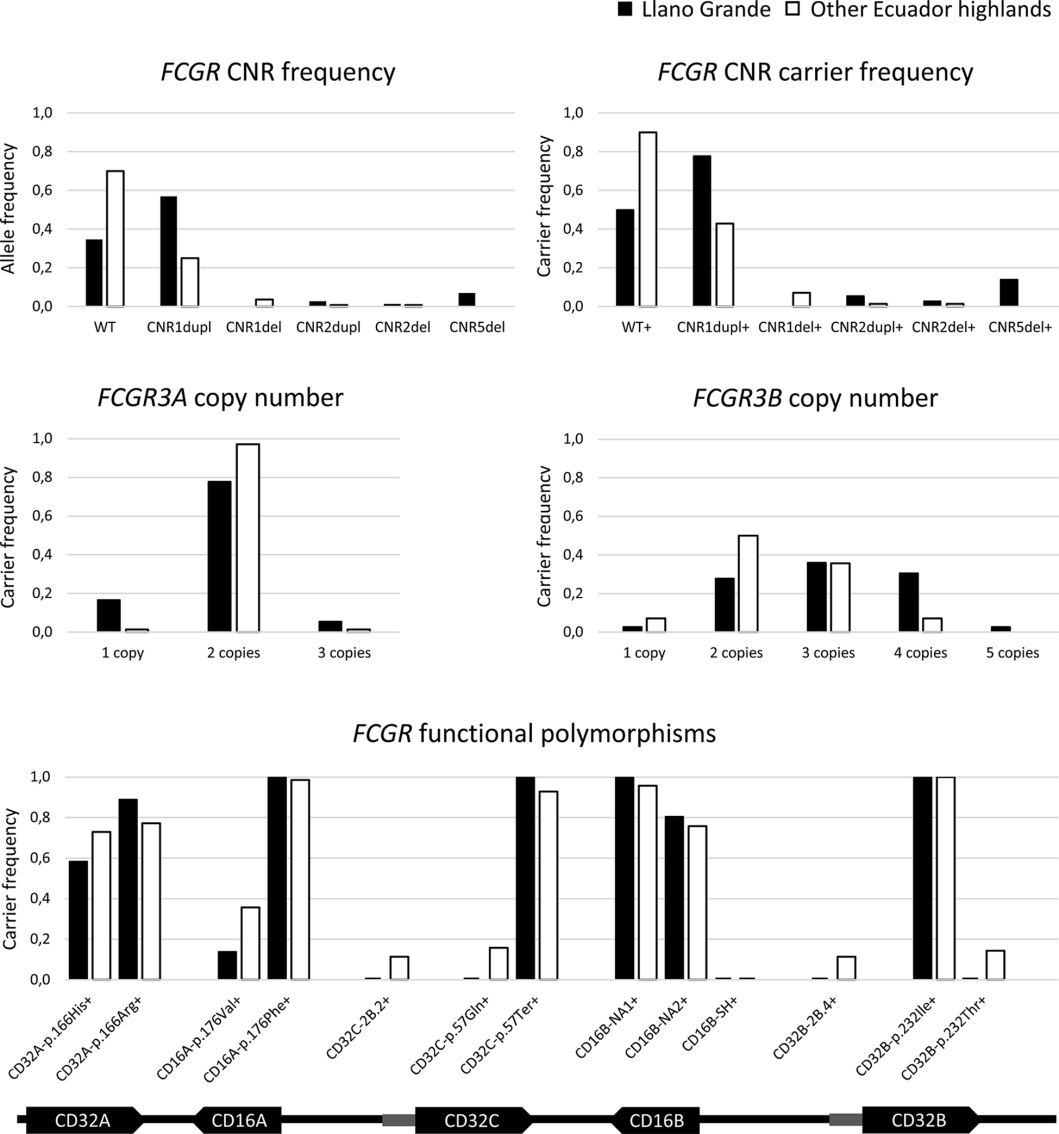
*FCGR* genetic variation in highlands Ecuadorians. Additional details are provided in **Supplementary Table 3**. Any putative CNR4 duplication is indistinguishable from and classified as CNR1 duplication, in agreement with all previous reports. Hypothetical rare combinations of CNR1 duplication on one chromosome and CNR1 deletion on the other one of the same donor are indistinguishable from and would have been classified as WT/WT. *FCGR3B/A* was included in neither *FCGR3B* nor *FCGR3A* frequencies.

As hypothesized, we found five additional carriers of the CNR5-deletion associated with the novel hybrid gene *FCGR3B/A* (**Figure 3** and **Supplementary Table 1**). In contrast, haplotypes with complete *FCGR3A* deletion were not overrepresented in comparison to other populations with different ethnic backgrounds (**Supplementary Tables 1** and **2**) – a single individual was heterozygous for CNR2 deletion. Overall, six individuals (16.7%) carried a single copy of a functional *FCGR3A*, but no new complete CD16A deficient individuals were found.

*FCGR3* intron 3 sequence analyses of the newly identified CNR5 deletion carriers showed identical break-point regions to the one we described in the first family members, suggesting that they all inherited the result of the same genetic event. Based on this information, we designed a PCR-SSP screening method that targets a combination of SNPs unique to *FCGR3B/A*, producing an 1178-bp specific amplicon that encompasses the recombination region (**Supplementary Figure 1**). PCR-SSP assays of positive and negative controls agreed with sequencing data. We expect this method to enable quick, simple and specific search for further *FCGR3B/A-*CNR5 deletion carriers in other populations.

The distribution of CNV and functional polymorphisms affecting the *FCGR* locus in Llano Grande significantly diverged from other populations (**Supplementary Tables 1** and **3**). Of note, less than 20% of Llano Grande donors carried the canonical configuration of the *FCGR* locus on both chromosomes. More than three quarters of them (77.8%) carried at least one CNR1 duplication (2X *FCGR2C-FCGR3B*), almost a third (30.6%) presenting it on both chromosomes (**Figure 3** and **Supplementary Table 2**). Of note, two individuals seemed to carry tandem CNR1 duplications (i.e. three or four copies of that region) on the same haplotype, in line with a previous report (3). Conversely, we found no example of the reciprocal CNR1 deletion among the 36 individuals from Llano Grande. Given the unusually high frequency of individuals with four *FCGR3B* (CD16B) gene-copies, due to CNR1 duplication, MLPA results were further confirmed using a restriction enzyme digest variant ratio (REDVR) assay (46). REDVR analyses (not shown) of six samples with four *FCGR3B* gene-copies, along with appropriate controls with 1–3 gene copies, were all consistent with MLPA results.

Assessment of functional *FCGR* SNPs (**Table 1**) revealed that the *FCGR2C*-p.57Ter variant, which abolishes CD32C expression, was present in all individuals, none of them having a single functional *FCGR2C* allele. Similarly, no individual carried the *FCGR3A*-176V/V genotype associated with higher CD16A affinity for IgG1 and IgG3, most of them (77.8%) being homozygous for the lower-affinity p.176Phe allele. Moreover, only four individuals (11.1%) carried the higher-affinity *FCGR2A* (CD32A)-166H/H genotype. In *FCGR2B*, no individual carried the p.232Thr allele, which impairs cell surface expression of the inhibitory CD32B receptor. Likewise, we found no examples of the promoter haplotypes *FCGR2B* 2B.4 (c.−386C/−120A), associated with increased CD32B expression, and *FCGR2C* 2B.2 (c.− 386C/−120T) (**Figure 3** and **Supplementary Table 3**). Finally, the *FCGR3B*-NA1 haplotype more common in South Americans and Asians than in Europeans and Africans (4, 5, 29, 31, 47–51), shows its highest frequency in Llano Grande – all individuals carried at least one copy; and none of them presented the *FCGR3B*-SH haplotype (**Figure 3**).

### High Frequency of *FCGR* CNV in Individuals From Ecuador Highlands

Considering the high frequency of *FCGR* genetic variants found in Llano Grande, we next sought to assess whether peculiarities in the distribution of these polymorphisms are generally shared by other Ecuadorian highlands populations. To that end, we studied the diversity of the *FCGR* locus in another sample of 70 healthy individuals from Ecuador highlands.

A complete report on the frequencies of all studied genetic variations is presented in **Supplementary Tables 1–3**. Resembling at lower frequency what we saw in individuals from Llano Grande, three and four copies of *FCGR3B* were found in 35.7% and 7.1% of donors, respectively, all of them estimated to correspond to CNR1/4 duplications in one or both chromosomes (**Figure 3** and **Supplementary Tables 1, 2**). These frequencies are similar to those previously reported in South American and Asian populations and significantly higher than those of Europeans and Africans (2, 4, 29, 30, 40, 46, 52). In contrast, only five individuals (7.1%) were *FCGR3B*-hemizygous due to CNR1 deletion, its low frequency being in line with those of populations with diverse backgrounds (**Figure 3** and **Supplementary Table 1**).

As in other populations (2, 4, 5, 29, 30, 40, 46, 52), *FCGR3A* was scarcely affected by CNV, with low frequencies of both CNR2 deletion and duplication, and absence of CNR3 and CNR5 variations, the latter in sharp contrast to the Llano Grande population. CNR4 deletions were also absent (**Figure 3** and **Supplementary Table 1**).

*FCGR2A-*p.His166Arg and *FCGR2C*-p.Gln57Ter genotype frequencies were similar to those found in other populations with diverse ethnic backgrounds. As in Llano Grande, we found a particularly high frequency of *FCGR3B*-NA1 haplotype carriers (95.6%), and no *FCGR3B*-SH haplotypes (**Figure 3** and **Supplementary Table 3**). Moreover, the *FCGR3A*-176V/V genotype was also underrepresented in Ecuador highlands compared to Europeans (p=0.009) (29) and most other populations; and no individual carried a *FCGR2B-*232T/T genotype (**Figure 3** and **Supplementary Table 3**). Finally, *FCGR2B*-2B.4 and *FCGR2C*-2B.2 promoter haplotype frequencies in highlands Ecuadorians were significantly higher than in Chinese (p<0.001) and moderately lower than in Europeans (p= 0.085 and p=0.035, respectively) (31) (**Figure 3** and **Supplementary Table 3**).

### Strong Linkage Disequilibrium Across the *FCGR* Locus in Ecuadorians

**Figure 4:**
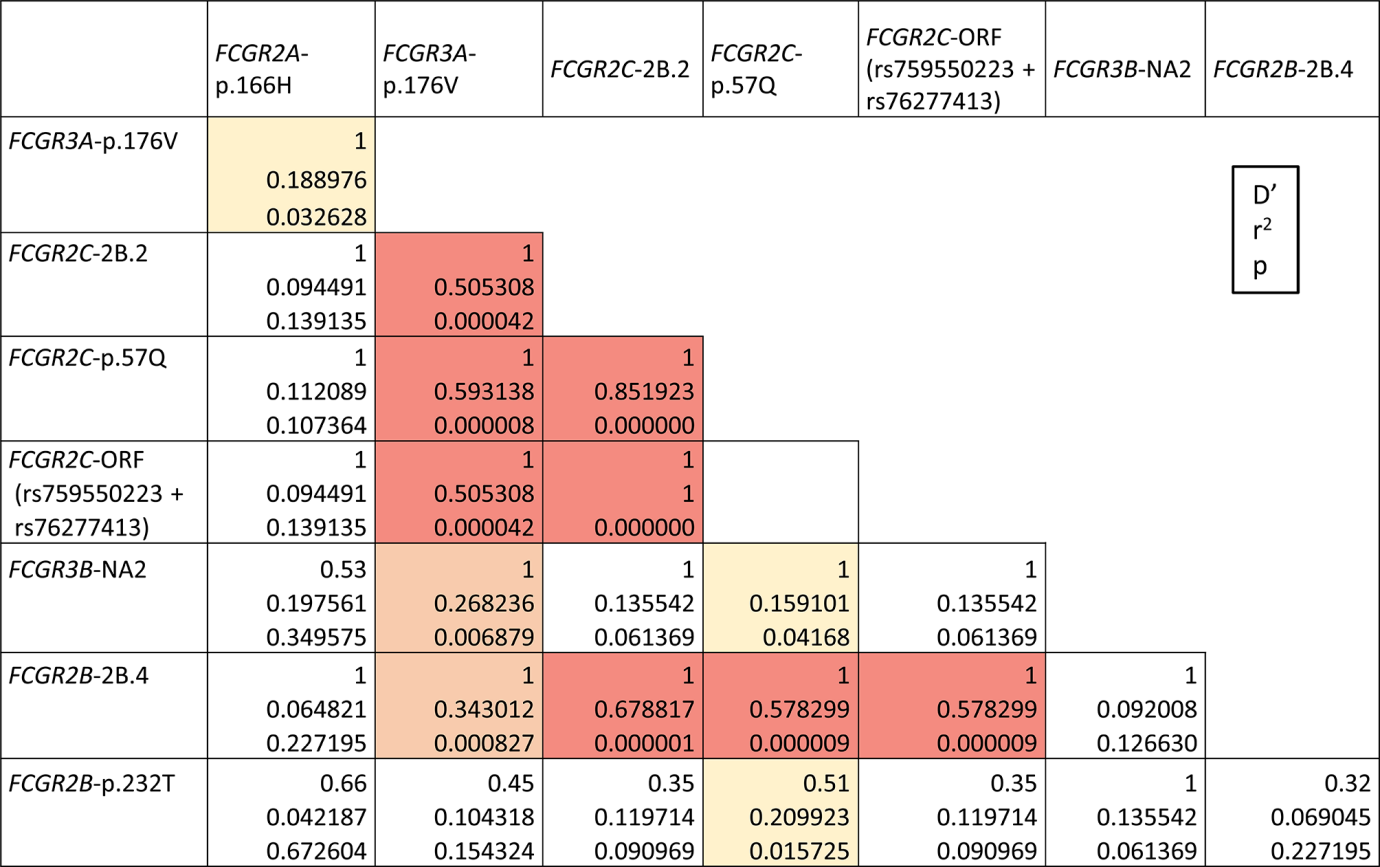
*FCGR* functional variants linkage disequilibrium in highlands Ecuadorians (Quito and Cuenca, N=35). LD combinations with p<0.0001 are highlighted in red; other statistically significant LD combinations are highlighted in orange (p<0.01) or yellow (p<0.05). All shown LDs are positive. Only individuals without CNV in the *FCGR* locus are analysed here. Combined SNP-CNV analysis is shown in **Supplementary Tables 3** and **4**.

Strong LD within *FCGR* locus has been reported in ethnically diverse populations, most of the data relating the *FCGR2C*-ORF allele with variants of *FCGR2A*, *FCGR3A* and *FCGR2B* (4, 31–33). Our results reveal similar associations in our second study-population from Ecuador highlands (**Figure 4**). In particular, we found strong LD of the classic *FCGR2C*-ORF haplotype (determined by p.57Q at rs759550223 and a consensus sequence at rs76277413) with *FCGR2C*-2B.2 (D’=1, r^2 =^ 1) and *FCGR2B*-2B.4 (D’=1, r^2 =^ 0.58) haplotypes, and with *FCGR3A*-176V (D’=1, r^2 =^ 0.51). Likewise, *FCGR3A*-p.176V was in strong LD with both *FCGR2C*-2B.2 and *FCGR2B*-2B.4 (D’=1, r^2 =^ 0.51 and D’=1, r^2 =^ 0.34) and with *FCGR3B*-NA2 (D’=1, r^2 =^ 0.27). Weaker LD of *FCGR3A*-p.176V with *FCGR2A-*p.166H and of *FCGR2C*-ORF with *FCGR3B*-NA2 and *FCGR2B*-p.232T were detected too. No statistically significant LD was found between CNR1 variations and flanking SNPs, except for negative LD of CNR1 duplication with *FCGR2B*-p.232I (p=0.02, **Supplementary Table 4**).

Complete lack of most of those genetic variants (*FCGR2C*-ORF+2B.2, *FCGR3A*-p.176V, and *FCGR2B*-2B.4) in Llano Grande donors precluded determination of D’/r^2^ values, but it also indirectly confirms that they constitute a conserved extended haplotype, absent in this population, but seen in other Ecuadorians (e.g. 8.57%, in our highlands sample, or up to 15.71% if parts of the haplotype are considered). Weak LD was observed between CNR5 deletion and *FCGR2A*-p.166H (p=0.04, **Supplementary Table 4**).

### *FCGR3* Genetic Variation Modifies Receptor Expression Levels

**Figure 5:**
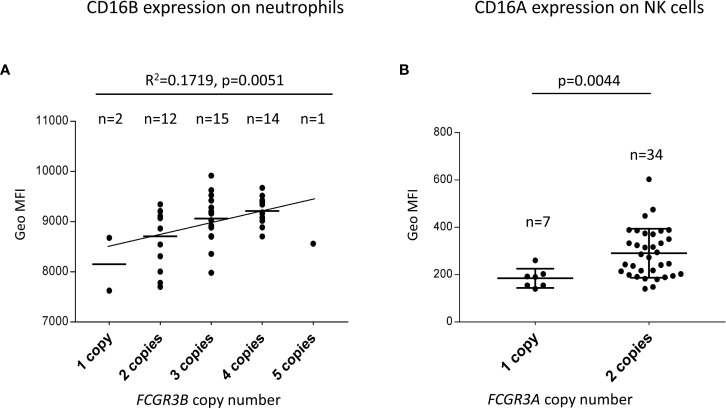
*FCGR3B* and *FCGR3A* copy-number modifies receptor expression. **(A)** A linear regression test predicts a direct correlation between *FCGR3B* copy number and CD16B surface expression on granulocytes. Additional tests were performed to confirm that the relationship between predictor and outcome is linear, the error variance is constant and the errors are normally distributed and independent. **(B)** *FCGR3A* two-copy carriers express significantly more CD16A on NK cells than hemizygotes (a Mann-Whitney non parametric test was used to compare CD16A MFI on NK cells between the two groups).

CNV of both *FCGR3A* and *FCGR3B* has been reported to influence receptor cell surface density (29, 40, 53–55). However, the relatively low frequency of many such genetic variants limited validity of those observations. To further characterize the functional/phenotypic consequence of the increased CNV observed in Llano Grande, we performed flow cytometry analysis on blood samples from 44 Llano Grande volunteers. Our results confirm that increasing copy-number is directly related to increased CD16B and CD16A expression on granulocytes and NK cells, respectively (**Figure 5**). The gene dosage effect observed for CD16B, though statistically significant (R^2 =^ 0.172, p=0.005), appears weaker than in previous reports (29, 55). This is mostly because the only carrier of five *FCGR3B* copies did not show particularly high expression levels, breaking linearity of the distribution; unfortunately, re-bleeding for repeated test was unfeasible. Exclusion of this outlier would yield an R^2^ value of 0.232 (p=0.001).

## Discussion

Previous studies reported high functional polymorphism along the *FCGR* locus, which is subjected to ethnic variation and has been shown to increase predisposition to disease (2, 4, 5, 29, 30, 40, 46, 52). These variations include fairly frequent CNV resulting from deletion or duplication of a small number of well-defined CNRs. We have recently found a new 82.5 kb deletion in the *FCGR* locus, which we name CNR5, associated with CD16A and CD16B deficiency and formation of a chimeric *FCGR3B/A* gene (34). In this study we further characterize this novel deletion, and provide information on the organization, genetic variation and LD within the *FCGR* locus in two Ecuadorian populations.

The high degree of sequence identity between *FCGR* genes facilitated recurrent events of duplication and recombination. For instance, both chimeric *FCGR2A/C*, with decreased FcγRIIa expression and function, and *FCGR2C/A*, with increased protein expression, have been described (3). Furthermore, ectopic CD32B expression on NK cells or *FCGR2B*-null alleles were also found as a result of unequal crossover (3, 6, 30). Likewise, the novel CNR5 deletion described herein results in a hybrid *FCGR3B/A* gene. We show that CNR5 limits lay at the beginning of intron 3 of *FCGR3A* and *FCGR3B* genes and thus the resulting chimeric protein would carry a promoter region and a membrane-distal Ig-like domain matching those of CD16B, whilst the membrane-proximal Ig-like domain and the rest of its structure are alike to CD16A. Our results confirm that this recombinant *FCGR3B/A* gene is readily transcribed, and that the ensuing hybrid protein can be expressed on the cell surface, but only in presence of adaptor FcϵR1γ or CD247 molecules. However, we were unable to demonstrate expression of this recombinant CD16B/A protein in granulocytes by either flow cytometry or western blot, possibly owing to very low expression levels.

Previous assessment of *FCGR* structure in several large cohorts with genetically diverse backgrounds documented ethnic variations (3, 31). However, only smaller samples of Amerindian populations have been studied (2, 52). Thus, the possibility that the novel *FCGR3B/A* recombination event might be characteristic for some South American populations could explain why it has not been described in previous studies of other populations. Indeed, our frequency study in Ecuadorians found an additional five CNR5 deletion carriers. Also supporting such perspective is the high prevalence of *FCGR3B*-NA1 haplotype in South Americans, which may have favoured its recombination with *FCGR3A* to generate CNR5 and *FCGR3B/A*. Alternatively, the very high degree of similarity between the hybrid *FCGR3B/A* and the parental *FCGR3A* and *FCGR3B* genes could have precluded its identification by some methods used for CNV estimation and thus its frequency might have been underestimated. Indeed, methods commonly used to assess CNV at *FCGR* locus other than MLPA cannot accurately identify CNR5 variations; Paralogue Test Ratio (PRT) described by Niederer et al. (5) would wrongly classify CNR5 deletions (or duplications) as CNR1 variations; and the combination of PRT and REDVR described by Hollox et al. (46) would probably classify them as “discrepant genotypes” (because in the samples carrying a CNR5 deletion REDVR assay would estimate a *FCGR3A* deletion, while PRT would determine a *FCGR3B* deletion). CNR5 deletion was a frequent trait in Llano Grande, however we found no homozygous carriers in this study. Although this might relate to the relatively small sample size, alternative explanations include selective pressures favouring maintenance of *FCGR3A* in the genome. Supporting such perspective is that a single family of immunodeficient individuals completely lacking *FCGR3A* has been described to date, and gene absence in those cases resulted from combination of two different genetic events. Homozygous CNR5 deletion would imply complete lack of both *FCGR3A* and *FCGR3B* (as well as *FCGR2C*, non-functional in most individuals), limiting the FcγR family of low-affinity receptors to CD32A, CD32B and the chimeric, possibly non-expressed CD16B/A. Although complete CD16B and CD32C deficiencies due to CNR1 deletion are rather common and have been described in otherwise healthy donors, further studies are warranted to clarify the consequence of homozygous CNR5 deletion on human health.

As it is the case with other South American native groups, the genetic structure of the Llano Grande population has likely been affected by inbreeding and genetic drift (35). In contrast, the other population of highlands Ecuadorians appears to have undergone more genetic admixture, as we observed a higher degree of *FCGR* diversity than in Llano Grande. Nevertheless, comparison with other ethnically diverse populations unveils clear characteristic features of the *FCGR* locus in native South Americans (**Supplementary Tables 1** and **3**). Of note, we could confirm the high frequency of both CNR1 duplication and the *FCGR3B*-NA1 haplotype, previously reported in this geographical region (2, 47, 48, 52, 56). The increased *FCGR3B* copy-number, concomitant to CNR1 duplication, possibly confers augmented neutrophil adhesion and immune complex uptake; similarly, common finding of the higher-activity CD16B-HNA-1a variant could derive from selective pressures favouring immune complex clearance by high phagocytosis variants, most probably related to infectious disease burden, as previously suggested (2, 55). Alternatively, the recently described CD16B function as a negative regulator of neutrophil-mediated ADCC (16) could be particularly relevant in a population where more than three quarters of its members carry three or more copies of *FCGR3B* and deserves further attention.

None of the studied individuals from Llano Grande carried the *FCGR3A*-176V/V genotype, associated with an increased affinity for IgG1 and IgG3, and its frequency was also low in highlands Ecuadorians. Similarly, the *FCGR2B* 2B.4 promoter haplotype, which increases the expression of the inhibitory CD32B was absent in Llano Grande and underrepresented in highlands Ecuadorians. It is of note that these genetic variants are associated in an extended haplotype (CD16A-176V – CD32C-ORF/2B.2 – CD32B-2B.4), absent in Llano Grande. This haplotype combines CD16A and CD32C allelic variants putatively associated with stronger activation of NK cells and monocyte/macrophages, with higher expression of the inhibitory receptor CD32B on several lymphoid and myeloid lineages. It is, therefore, not obvious what selective pressures favoured such combination. In Europeans, in whom this haplotype is found in ~20% of individuals, it is tempting to speculate that these allotypes evolved together to ensure a balance between strong activating (CD16A-176V and CD32C-ORF/2B.2) and inhibitory (CD32B-2B.4) FcγR in response to an infectious burden, favouring increased ADCC and other IgG-mediated responses (30, 57, 58). In this context, different pathogen burdens in South America might have selected alternative genetic variants, like CD16B-HNA-1a, and it is possible that differences in FcγR genetics between different ethnic groups may confer diverse susceptibility to disease and influence responsiveness to vaccination and immunotherapy.

The current MLPA method for the study of *FCGR* should readily identify hypothetical, yet undiscovered CNR5 duplications reciprocal to the CNR5 deletion we have characterized. Such duplication would generate a hybrid *FCGR3A/B* gene, predicted to be expressed on NK cells and monocytes as a GPI-anchored CD16. The fact that we found no such CNR5 duplications in highlands Ecuadorians has several possible explanations. Firstly, the small sample size analysed in the current study, which calls for larger cohort research to identify and possibly establish the frequency of that genetic event. Also worth considering is the possibility that putative CNR5 duplications might have gone overlooked in previous studies of *FCGR* genes, because many of these were performed with methods of lower resolution than MLPA – in this regard, the elevated *FCGR3B* copy number observed in South Americans (2, 52) might be concealing some CNR5 duplications. Finally, it is also possible that any selective pressures that allowed or favoured persistence of the CNR5 deletion in the population, might have selected against its reciprocal duplication, reducing its frequency or extinguishing it altogether.

In summary, we provide a molecular characterization of a novel CNR deletion of clinical consequence in the *FCGR* locus, establishing its recombination point and the expression pattern of the ensuing chimerical product. Furthermore, we have determined its frequency among healthy subjects of the same population, and extended current evidence of ethnic variation in the *FCGR* locus, showing key characteristics of its structure and diversity in two Andean populations native from Ecuador highlands. Whether the distinctive genetic features of these are due to natural selection remains an open question of potential clinical interest.

## Data Availability Statement

All relevant genetic polymorphism data is contained within the article. Novel *FCGR3* sequences were submitted to GenBank (accession nos. MW620867, MW573976 and MW573977).

## Ethics Statement

The studies involving human participants were reviewed and approved by *Comité Ético de Investigación con Medicamentos, Hospital Universitario Puerta de Hierro, Majadahonda* (no. 13.18). The patients/participants provided their written informed consent to participate in this study.

## Author Contributions

MM designed and performed experiments, analysed and interpreted data, and wrote the manuscript. AP-P performed experiments, analysed and interpreted data, and revised the manuscript. KA-S and AB-M performed experiments and analysed and interpreted data. AA-V contributed samples from Ecuador Highlands. HTR designed the study, directed research, and critically revised the manuscript. CV designed the study, directed research, and wrote the manuscript. All authors contributed to the article and approved the submitted version.

## Funding

This work was funded by grant SAF2016-80363-C2-2-R to CV, grants SAF2014-58752-R and SAF2017-83265-R to HTR, and grant PI-18-00720 to AA-V from the Spanish *Ministerio de Ciencia e Innovación* (AEI/FEDER, EU). Publication costs were defrayed by a nominative grant from *Consejería de Sanidad de la Comunidad de Madrid* to support research in *Fundación de Investigación Biomédica Puerta de Hierro*. KA-S was supported by grant PEJ-2017-AI/BMD-7377, with co-financing by EU Youth Employment Initiative, European Social Fund (91.89%), and *Consejería de Educación, Juventud y Deporte de la Comunidad de Madrid*. MM is supported by the *Asociación Española contra el Cáncer* Foundation grant GCB15152947MELE.

## Conflict of Interest

The authors declare that the research was conducted in the absence of any commercial or financial relationships that could be construed as a potential conflict of interest.
