# Supplementary figures and images for: FCGR Genetic Variation in Two Populations From Ecuador Highlands—Extensive Copy-Number Variation, Distinctive Distribution of Functional Polymorphisms, and a Novel, Locally Common, Chimeric FCGR3B/A (CD16B/A) Gene
Source: Front Immunol. 2021 May 24;12:615645. doi: 10.3389/fimmu.2021.615645 (PMC8183472; doi:10.3389/fimmu.2021.615645)

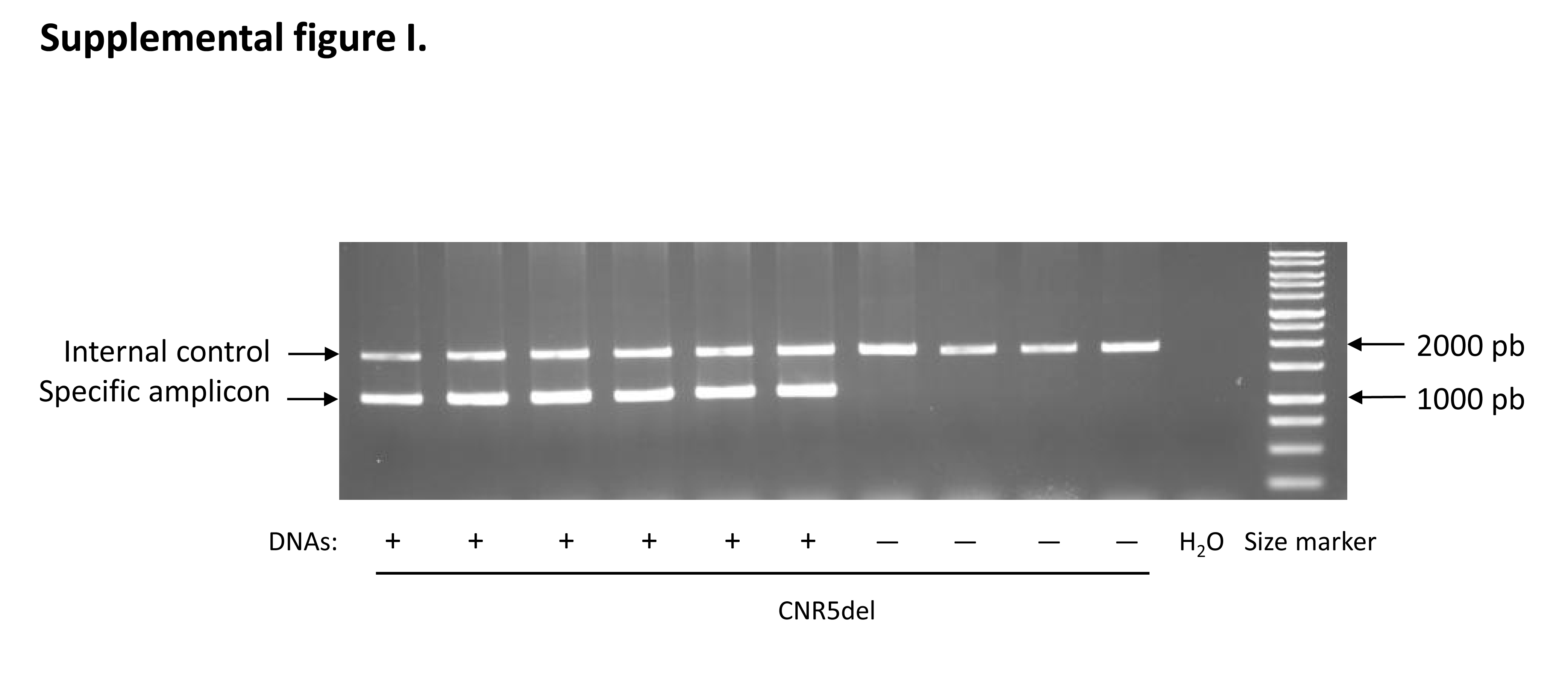

Supplement: Supplementary file 2 [file Image_1.tif]
